# Alzheimer’s Staffing, Services, and Outcomes in Adult Day Health Centers

**DOI:** 10.1093/geroni/igaa057.270

**Published:** 2020-12-16

**Authors:** Joanne Spetz, Jason Flatt

**Affiliations:** 1 University of California, San Francisco, San Francisco, California, United States; 2 University of Nevada, Las Vegas, School of Public Health, Las Vegas, California, United States

## Abstract

Growing demand for care for Alzheimer’s Disease and related Dementia (ADRD) has resulted in rising use of adult day health centers (ADHCs), which employ teams of professionals including licensed nurses, nursing aides, social workers, and activity directors. This study evaluates the scope of services and staffing models of ADHCs that provide care to persons with ADRD compared to ADHCs that do not, and examines whether there is an association between staffing and client outcomes, measured as rates of hospitalizations, falls, and emergency department visits. We used facility-level data from the 2014 National Study of Long-Term Care Providers (NSLTCP) Adult Day Services Center module. We conducted bivariate comparisons and estimated multivariate regressions to identify ADHC characteristics associated with staffing and client outcomes. ADHCs that offered ADRD services had higher average daily attendance, greater shares of revenue from Medicaid and self-payment, and greater proportions of Blacks and females. They also had greater percentages of enrollees with depression, cardiovascular disease, diabetes, and needing assistance with activities of daily living. There were also greater numbers of registered nurse, licensed practical nurse, and social worker hours per enrollee day, but fewer activity staff hours per enrollee day. Multivariate regressions focused on ADHCs that offered skilled nursing services and revealed that total staff hours per enrollee day were not higher in ADHCs that provided ADRD services, controlling for other characteristics. However, staffing was greater in chain-affiliated ADHCs. Higher staffing levels were associated with lower rates of falls and emergency department visits.

